# Overlapping but Divergent Neural Correlates Underpinning Audiovisual Synchrony and Temporal Order Judgments

**DOI:** 10.3389/fnhum.2018.00274

**Published:** 2018-07-03

**Authors:** Scott A. Love, Karin Petrini, Cyril R. Pernet, Marianne Latinus, Frank E. Pollick

**Affiliations:** ^1^School of Psychology, University of Glasgow, Glasgow, United Kingdom; ^2^Physiologie de la Reproduction et des Comportements, Institut National de la Recherche Agronomique, Centre National de la Recherche Scientifique, IFCE, Université de Tours, Nouzilly, France; ^3^Department of Psychology, University of Bath, Bath, United Kingdom; ^4^Brain Research Imaging Centre, Imaging Sciences, University of Edinburgh, Edinburgh, United Kingdom; ^5^UMR1253, iBrain, Université de Tours, Inserm, Tours, France

**Keywords:** multisensory, audiovisual, fMRI, temporal processing, asynchrony

## Abstract

Multisensory processing is a core perceptual capability, and the need to understand its neural bases provides a fundamental problem in the study of brain function. Both synchrony and temporal order judgments are commonly used to investigate synchrony perception between different sensory cues and multisensory perception in general. However, extensive behavioral evidence indicates that these tasks do not measure identical perceptual processes. Here we used functional magnetic resonance imaging to investigate how behavioral differences between the tasks are instantiated as neural differences. As these neural differences could manifest at either the sustained (task/state-related) and/or transient (event-related) levels of processing, a mixed block/event-related design was used to investigate the neural response of both time-scales. Clear differences in both sustained and transient BOLD responses were observed between the two tasks, consistent with behavioral differences indeed arising from overlapping but divergent neural mechanisms. Temporal order judgments, but not synchrony judgments, required transient activation in several left hemisphere regions, which may reflect increased task demands caused by an extra stage of processing. Our results highlight that multisensory integration mechanisms can be task dependent, which, in particular, has implications for the study of atypical temporal processing in clinical populations.

## Introduction

Temporal correspondence is a key principle of multisensory integration, thus manipulating the relative timing of the unimodal components (e.g., audio and visual cues) of a cross-modal stimulus is one of the most extensively and widely used tools for investigating multisensory processing. The most commonly used tasks for studying temporal processing, whether in a unimodal or cross-modal context, are synchrony judgment (SJ) and temporal order judgment (TOJ) paradigms. During a SJ task, participants decide whether cues are in synch or out of synch, whereas in a TOJ task, they decide which cue came first (or last). Both tasks allow for the extraction of a point of subjective simultaneity (PSS) and a temporal integration window (TIW) to index temporal processing ability. In their review, Keetels and Vroomen (2012) observed that SJs and TOJs have often been used interchangeably to investigate temporal processing, despite the fact that comparative studies report differences between the tasks. Indeed, accumulating behavioral evidence suggests that these tasks actually measure different processes, or at least different aspects of the same process, even within the same participant for the same stimulus (van Eijk et al., 2008; Vatakis et al., 2008; Fujisaki and Nishida, 2009; Petrini et al., 2010; Maier et al., 2011; Vroomen and Stekelenburg, 2011; Love et al., 2013). At the group level, the PSS derived from an audiovisual SJ task tends to be visual-leading, i.e., the onset of the visual cue needs to occur before the auditory cue for synchrony to be maximally perceived, whereas when it is derived from TOJ using the same stimuli and participants it is often found to be audio-leading (van Eijk et al., 2008; Petrini et al., 2010; Love et al., 2013). At the individual level, there is no correlation between the two tasks for either PSS or TIW (van Eijk et al., 2008; Vatakis et al., 2008; Fujisaki and Nishida, 2009; Vroomen and Stekelenburg, 2011; Love et al., 2013). Moreover, improved performance due to training on one of the tasks does not transfer to the other (Mossbridge et al., 2006). Here we aimed to investigate how these differences are manifested in brain activity by examining whether they reflect completely independent temporal processing networks, different levels of activity from the same network, or overlapping (share some mental processes and associated neural architectures) but divergent networks (require specific processes).

Several experiments have investigated the neural mechanisms involved in audiovisual SJs (e.g., Miller and D'Esposito, 2005; Lewis and Noppeney, 2010; Stevenson et al., 2010; Noesselt et al., 2012), but until very recently there was no evidence of the mechanisms involved in audiovisual TOJs, nor any direct comparison of the networks underlying these two tasks. Recently, however, using a simple beep-flash stimulus and an event-related functional magnetic resonance imaging (fMRI) analysis, Binder (2015) highlighted several left hemisphere regions (superior/inferior lobule, middle/inferior gyrus and lateral occipital cortex) that activate more during audiovisual TOJs than SJs. Similarly, but in response to unimodal tactile stimulation, Miyazaki et al. (2016) observed more activation for TOJs than SJs in left premotor cortex, left posterior parietal cortex, right premotor cortex and bilateral thalamus. In contrast, within left insular cortex they observed greater activation for SJs than TOJs. These studies were important and timely in reporting differences between TOJs and SJs, nevertheless many questions about the nature of these neural differences are still unanswered. For example, are the differences between TOJ and SJ only evident in overlapping brain regions or do they have divergent neural correlates? Are these differences present for more complex/natural audiovisual stimuli (i.e., stimuli for which we have accumulated prior experience about their visual and auditory correspondence)? Do these neural differences depend on similar or different processing time-scales?

The current study used a more complex audiovisual stimulus than in Binder (2015) of a point-light drummer (Petrini et al., 2009a,b; Love et al., 2013) to test the hypothesis that SJs and TOJs have different neural correlates in the human brain. Point-light drumming is formed by a visual and auditory continuous stream of information that is closer to the stimulation we receive in real life compared to simple beep-flash stimuli. Additionally, this stimulus represents a human action but at the same time is similar to Binder (2015) stimulus in terms of low-level characteristics (e.g., white dots appearing on a black background and absence of contextual information). The decision to use a more complex stimulus was necessary to understand whether the neural differences between SJ and TOJ are stimulus dependent as stimulus complexity is known to influence performance (Dixon and Spitz, 1980; Vatakis and Spence, 2006; Love et al., 2013; Stevenson and Wallace, 2013). This in turn would substantiate Binder (2015) findings with simple beep-flash stimuli by extending them to more complex and ecological situations. Indeed, we rarely experience events formed by one isolated visual and auditory stimulus rather we commonly experience complex audiovisual events formed by *streams* of visual and auditory events. Hence, it is essential to examine whether neural differences previously found with single-event simple stimuli extend to more complex everyday situations.

We used a mixed block/event-related design rather than a simple event-related design because research has demonstrated (e.g., Chawla et al., 1999; Donaldson et al., 2001; Visscher et al., 2003) that two different time-scales of neural activity can be investigated with fMRI: transient (event-related) and sustained (task/state-related) activity. Sustained effects are characterized by rises in the BOLD signal that plateau, or remain elevated, for a significant duration rather than quickly descending back to baseline as would a transient effect. This is an important distinction to make when exploring differences between two related tasks such as SJs and TOJs, as task differences could be explained by different sustained activity (“states-of-mind”) and/or transient trial-related activity (e.g., decision-making). Standard block and event-related designs pool (confound) these two different levels of processing and prevent their independent investigation (Donaldson, 2004), thus limiting our understanding of neural differences between SJ and TOJ tasks.

As both tasks are involved in temporal processing but show clear behavioral differences, it was predicted that the neural mechanisms underpinning these two tasks would be overlapping but also divergent. That is, that a network of task-independent temporal processing regions would be involved in both tasks along with other task-specific networks dependent on the judgment being made. Such an inherent divergence in the neural mechanisms underpinning the tasks should be evident regardless of the stimulus type being presented. Therefore, despite our use of a more complex stimulus than Binder (2015) convergent overall results between the two studies would be expected.

## Methods

### Experiment overview

Participants first completed a pre-fMRI behavioral experiment in which they made SJs and TOJs to synchronous and asynchronous audiovisual stimuli. Dependent on TOJ performance (R^2^ goodness-of-fit between data and fitted function) with the current stimulus, participants were classified, for that stimulus, into one of two groups: TOJ-able (*R*^2^ > 0.5) or TOJ-unable (*R*^2^ < 0.5). In the following fMRI experiment, participants made SJs and TOJs, but to a reduced stimulus set of synchrony conditions: individually defined task-specific PSSs, largest audio-leading (333 ms), largest video-leading (333 ms), and physically synchronous stimuli. Both TOJ-able and TOJ-unable participants were included in the fMRI experiment as statistical comparisons between these groups could be informative about how and why potential differences between the tasks occur.

### Participants

Twenty right-handed participants (10 female, mean age [range] = 24 [20–32]) took part. None had received any professional musical training, and all described themselves as “musical novices.” All had normal or corrected to normal vision and reported no hearing difficulties or any history of neurological disorders. All participants gave informed written consent in accordance with the Declaration of Helsinki and were paid for their participation. The University of Glasgow, College of Science and Engineering ethics committee approved the protocol.

### Stimulus preparation

**Figure 1:**
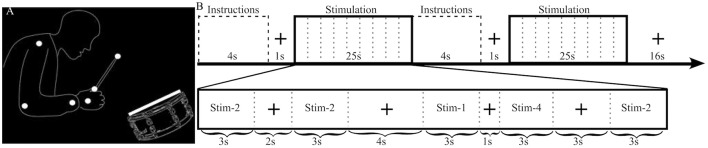
Stimulus illustration and experimental design. **(A)** Static sample frame of the jazz drummer dynamic point-light display. White dots represent the shoulder, elbow, wrist, hand, grip, and drumstick tip, while the white rectangle represents the drumhead. Faint outlines of the person and drum were not in the actual stimulus and are presented here to help illustrate what the point-lights represent. **(B)** The top row highlights the sequence of events and timing of the fMRI experimental design. The bottom row provides an example of the timing and contents of a stimulation block. In this example Stim-3 is presented 0 times, Stim-1 1 time, Stim-2 3 times, and Stim-4 1 time and each of the 4 possible fixation durations (1, 2, 3, 4 s) are presented in a random order.

The stimuli had previously been used in other studies, and a complete description of them can be found elsewhere (Petrini et al., 2009a,b; Love et al., 2013). They comprised dynamic audiovisual movies (3 s) containing the point-light representation of a drummer playing a swing groove at 120 beats per minute, with an accent on the second beat (Figure 1). Audio and visual cues were shifted relative to each other to produce stimuli with different cue onset asynchrony (COA). The video was shifted to begin either after the audio (−333, −267, −200, −133, and −67 ms) or before the audio (+333, +267, +200, +133, and +67 ms), producing a total of 10 asynchronous stimuli to be used in the pre-fMRI experiment. Negative and positive numbers will be used to refer to audio-leading and video-leading COA levels respectively, and 0 COA will refer to the synchronous condition. To prevent participants from having to stay in the MRI scanner for an uncomfortably long time only 4 COA levels were used during the fMRI experiment: two asynchronous (−333, +333 COA) and two “synchronous” (0 COA and the individually defined PSS). The −333, 0 and +333 COA conditions are provided as Supplementary Videos 1–3, respectively.

Stimuli were presented using MATLAB 2007b (MATHWORKS Inc., Natick, MA) and the Psychophysics Toolbox (Brainard, 1997; Pelli, 1997).

### Procedure and analysis of behavioral experiment

The behavioral session (~20 min) took place in a darkened room, where participants sat approximately 65 cm from a CRT monitor (FormacProNitron 19.380; 1,024 × 768 pixel resolution and 60 Hz refresh rate). Auditory cues were presented via Beyerdynamic DT headphones.

The two-alternative forced-choice experiment consisted of 20 blocks; in half of the blocks participants responded as to whether the audio and video cues were synchronous or not (SJ) and in the other half as to which cue came first (TOJ). Block order was randomized. Instructions describing whether a SJ or a TOJ was to be performed were displayed on screen for 4 s before the beginning of each block. There were 10 trials per COA level for each task. Written instructions emphasized the importance of understanding the difference between the two tasks and that it was crucial to be constantly aware of what the current task was.

Best-fitting Gaussian curves (least squares minimization using iterative maximum likelihood) to the number of synchronous and visual first responses were calculated for the SJ and TOJ data respectively. PSS was derived as the peak of the SJ probability function and as the 50% point of the TOJ cumulative distribution function, while the TIW was taken as the standard deviation of the Gaussian curve for each task (Petrini et al., 2010). Previous research has shown that some participants cannot successfully make TOJs (random responses or responses completely biased toward one response) for some stimulus types (Petrini et al., 2010; Love et al., 2013). In the current study, *R*^2^-values (goodness-of-fit between data and fitted function) below 0.5 were regarded as indicating that a participant could not successfully make the TOJ. This criterion was defined in our previous work as it corresponded well with subjective interpretations of what constituted good and bad data fits (Love et al., 2013). Furthermore, it represents a quantitative, replicable criterion that can be used and compared across experiments.

### Procedure and analysis of fMRI experiment

The fMRI procedure was similar to the behavioral experiment, except that a reduced stimulus set was presented: −333, 0, PSS, and +333. The PSS values were obtained individually from the pre-fMRI experiment separately for SJ and TOJ. To be as accurate as possible the individual PSS conditions were selected as the closest COA level to that of the PSS value derived from the pre-fMRI data fits. Although COA levels in the pre-fMRI experiment were restricted to ±333, ±267, ±200, ±133, ±67, and 0 ms, COA values for the PSS condition in the fMRI experiment could be any COA level between 0 and ±333 in 16 ms increments i.e., one frame at a time. This use of an individually determined stimulus level (PSS) is similar to the approach used by Binder (2015) to determine stimuli levels, but did not use the simultaneity threshold approach based on separate sound-first and flash-first trials. For TOJ-unable participants, we used average results from a behavioral study using identical stimuli (Petrini et al., 2010). An optimized mixed block/event-related design was used to enable investigation of differences between the tasks at both transient and sustained levels of processing.

Each of two functional runs (~22 min each) consisted of 32 stimulation blocks (half SJ and half TOJ, randomized) and after every two stimulation blocks there was a 16 s fixation block (Figure 1). Within a stimulation block (25 s) there were 9 events: 5 stimuli (each 3 s) separated by 4 fixation events (1, 2, 3, or 4 s in pseudorandom order). Each COA condition was presented a total of 40 times (20 per run) per task. To minimize the correlation between the transient (stimuli) and sustained (stimulation block) regressors the number of times an individual COA condition was presented within a single stimulation block was manipulated as follows: in a run, a COA level was presented 0 times during 4 stimulation blocks, once in 6 blocks, twice in 4 blocks and 3 times in 2 blocks, i.e., a total of 20 presentations for each COA level and task. One thousand sequences with different randomizations of the order of events and blocks were created and the best chosen by balancing efficiency and correlation. In the chosen sequence, the mean correlation between sustained and transient regressors was 0.47, which enabled reliable estimation of both types of BOLD response (Otten et al., 2002).

Auditory stimuli were presented via Sensimetrics S14 insert headphones at approximately 85 dB. The visual cue was back-projected (Panasonic PT-D7700E DLP; 1,024 × 768 pixel resolution, 60 Hz refresh rate) onto a screen behind the participant's head, visible via a mirror mounted on the MR head coil with an approximate viewing distance of 65 cm.

Functional images covering the whole brain (field of view: 210 mm, number of slices: 32, voxel size: 3 × 3 × 3 mm) were acquired with a 3T Tim Trio Scanner (Siemens) and a 32-channel head coil using an echoplanar imaging (EPI) sequence (ascending-interleaved, TR: 2 s, TE: 30 ms, flip angle: 77°). At the end of the fMRI session, high-resolution T1-weighted images (anatomical scan) were obtained (field of view: 256 mm, number of slices: 192, voxel size: 1 × 1 × 1 mm, flip angle: 9°, TR: 1.9 s, TE: 2.52 ms).

SPM8 software (Wellcome Department of Imaging Neuroscience, London, UK) was used to pre-process and analyse the imaging data. First, the anatomical scan was AC-PC centered; this correction was then applied to all EPI volumes. Functional data were slice-time corrected and subsequently motion corrected using a two-pass six-parameter rigid-body spatial transformation (Friston et al., 1996), which realigned all functional volumes to the first volume of the scan closest to the anatomical scan, and subsequently realigned all the images to the mean volume. The anatomical scan was co-registered to the mean volume and segmented. The functional and anatomical images were then normalized to the Montreal Neurological Institute (MNI) template using the parameters issued from the segmentation, keeping the voxel resolution of the original scans (3 × 3 × 3 and 1 × 1 × 1 mm respectively). Functional images were smoothed with an 8 × 8 × 8 mm full width at half maximum Gaussian kernel. Global linear trends and rapid aliased noise were minimized through high-pass filtering the data with a cutoff period of 128 s and an autoregressive [AR(1)] filter during statistical model estimation. All the analyses were conducted in a masked skull-stripped search volume, created by combining three tissue maps (white and gray matter and cerebrospinal fluid) output at the segmentation procedure.

Data were analyzed in a two-level random-effects analysis, with each run entered as a separate session. The first-level analysis involved a design matrix with 18 regressors per session. There were 10 regressors of interest: two for sustained-effects and eight for transient-effects (4 conditions × 2 tasks). SJ and TOJ sustained-effects were modeled using 25-s boxcar functions; transient-effects were modeled separately for each task and COA level with event-related impulse responses. Both the sustained and the transient regressors were convolved with a canonical hemodynamic response function. Eight regressors of no interest were included to account for the instruction periods, six realignment motion parameters and the grand mean.

Using the general linear model, parameter estimates for each regressor were calculated and used to create contrast images for a condition relative to baseline (Friston et al., 1995). The resulting images were used in repeated-measures ANOVAs conducted using the GLM Flex software (http://mrtools.mgh.harvard.edu/index.php/Main_Page#Welcome.21). The first ANOVA examined any differences in sustained-effects produced by the factors Task (SJ/TOJ) and Group (TOJ-able/TOJ-unable), plus their interaction. The second tested for differences in transient-effects produced by the factors Group (TOJ-able/TOJ-unable), Task (SJ/TOJ), COA Condition (−333/0/PSS/333), and their interactions. We report all clusters that were significant after multiple comparisons correction (*p* < 0.05) based on cluster-extent false discovery rate (Chumbley and Friston, 2009) with the auxiliary voxel-level threshold set at *p* < 0.0001.

## Results

### Behavioral results

**Figure 2:**
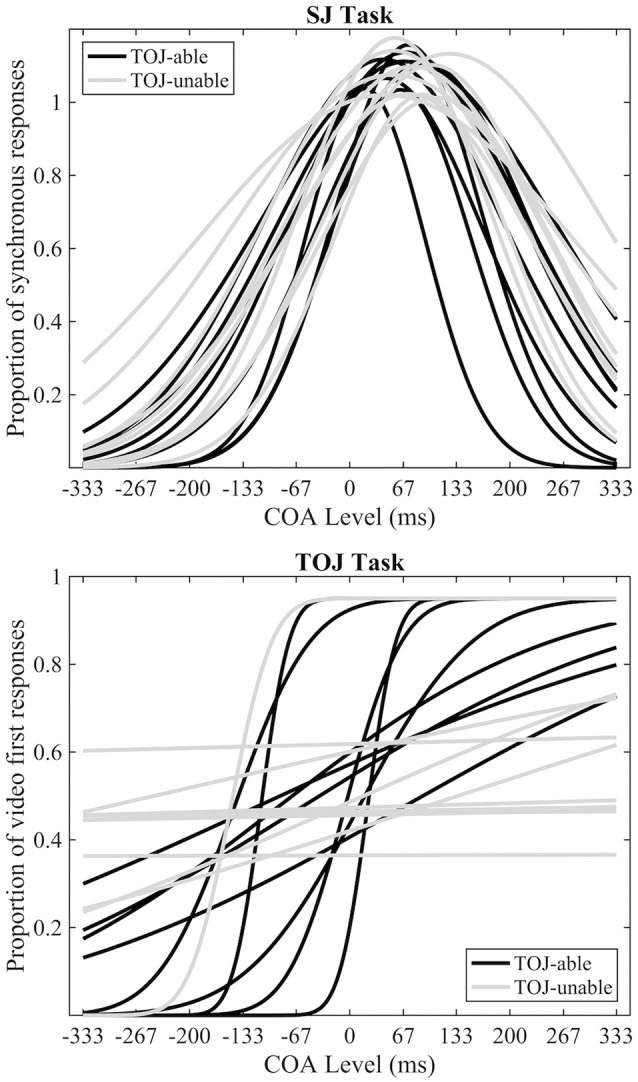
Pre-fMRI behavioral data. Individual best-fitting Gaussian functions for the SJ task **(top)** and individual best-fitting cumulative Gaussian functions for the TOJ **(bottom)** tasks. Black and gray functions represent TOJ-able and TOJ-unable participants respectively.

In line with previous research (Petrini et al., 2010; Love et al., 2013), data from the pre-fMRI experiment indicated that some participants could not successfully make TOJs (Figure 2). Eleven out of 20 participants were deemed unable to make TOJs based on an *R*^2^ value of < 0.5; from now on they will be referred to as the TOJ-unable group, with the other 9 participants being the TOJ-able group. The mean SJ PSS of all participants was a +70 ms (s.e.m = 5.7) video-leading stimulus, while the mean TOJ PSS from the TOJ-able participants was a −55 ms (s.e.m = 24.6) audio-leading stimulus. Paired-samples *t*-tests, using the pre-fMRI data of participants able to achieve both tasks (TOJ-able), highlighted a significant difference (*t*_8_ = 3.54, *p* = 0.008) between TOJ and SJ PSS but not TIW (*SJ* = 127 ms [s.e.m = 12], TOJ = 190 ms [s.e.m = 55], *t*_8_ = 1.243, *p* = 0.249). Comparison between the TOJ-able and TOJ-unable group using independent-samples *t*-tests indicated there was no difference in SJ PSS (difference 17.8 ms *t*_18_ = 1.628, *p* = 0.121) or TIW (difference 30.1 ms *t*_18_ = 1.759, *p* = 0.096) between the groups.

**Figure 3:**
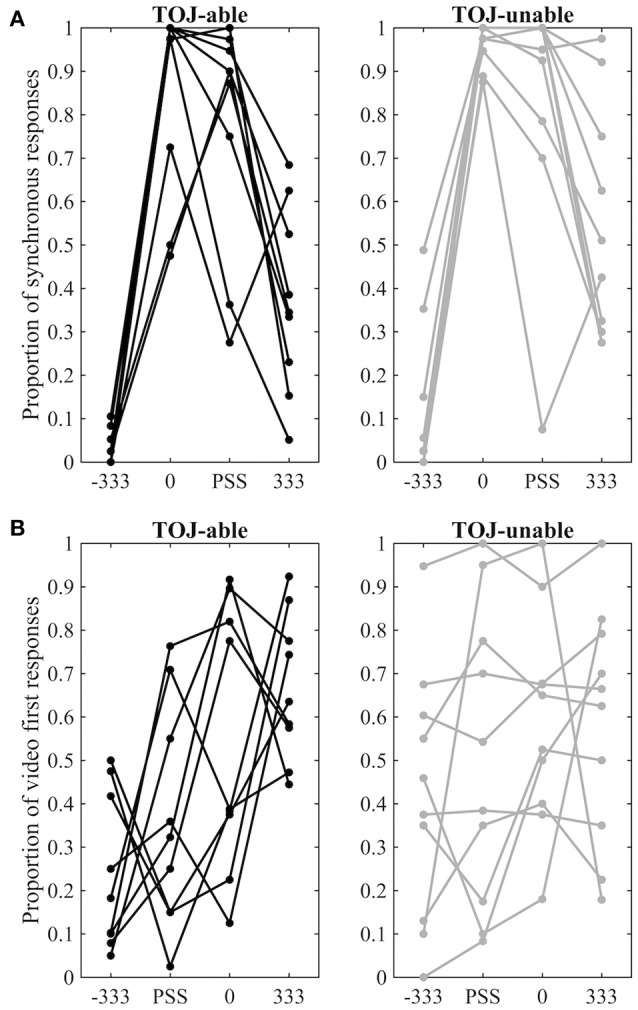
fMRI behavioral data. **(A)** Proportion of synchronous responses in the SJ task. **(B)** Proportion of video-first response in the TOJ task. Data points from different COA levels for the same individual are connected by lines. Data from TOJ-able participants are presented on the left with black dots and lines, while data from TOJ-unable participants are presented on the right with gray dots and lines.

Behavioral responses to the four conditions presented during fMRI scanning are displayed separately for each group and for each task in Figure 3. Furthermore, a direct comparison of individual performance inside and outside the MRI environment can be visualized in Supplementary Image 1. To compare behavior from pre-fMRI and during fMRI separate 3 Factor (Group: TOJ-able / TOJ-unable X Time: pre-fMRI/fMRI X COA: −333 0 and 333) repeated measures ANOVAs were conducted on the SJ and TOJ data. Both highlighted significant interactions between the Time and COA Factors [SJ: *F*_(2, 34)_ = 20.39, *p* < 0.001; TOJ: *F*_(2, 34)_ = 3.67, *p* < 0.036]. Visual inspection of the data showed that for both SJ and TOJ there was a difference in pre-fMRI and during fMRI performance but only for the +333 COA condition. For the SJ task this difference appeared as a higher proportion of synchronous responses during fMRI and for the TOJ task it appeared as a higher proportion of video-first responses during fMRI.

### fMRI results

**Figure 4:**
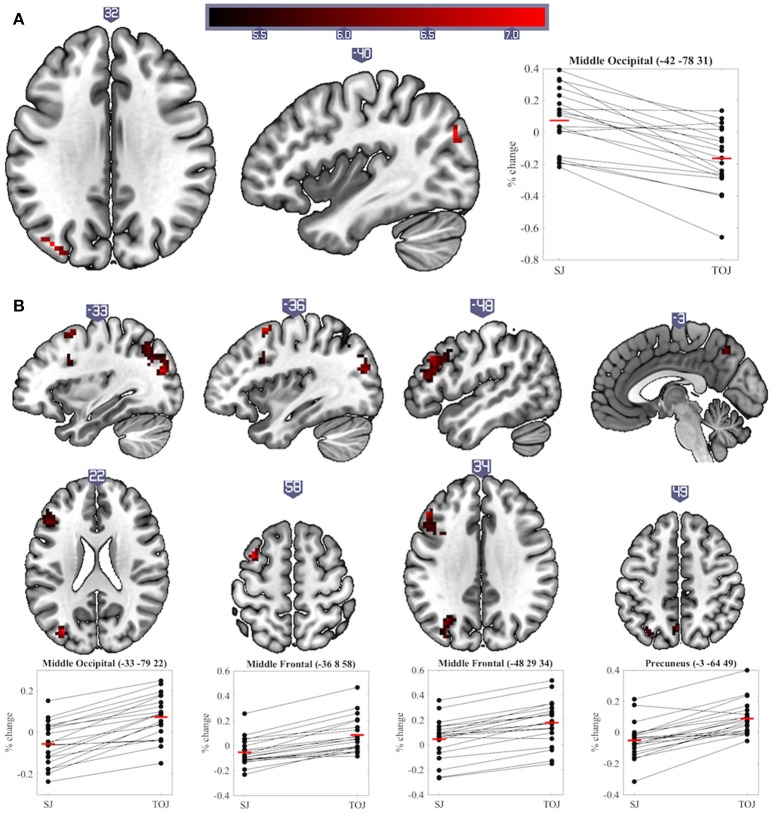
Significant clusters and individual participants' % signal change from the clusters peak voxel for **(A)** sustained and **(B)** transient main-effects of Task. % signal change was calculated using a scaling factor of 0.132 (Pernet, 2014). Clusters are presented on axial or sagittal slices of the MNI152_2009bet (Fonov et al., 2011) template using MRIcroGL (www.mccauslandcenter.sc.edu/mricrogl, 12/12/2012).

The main aim of this study was to explore differences in neural activity underpinning SJs and TOJs. Significant differences (*p* < 0.05 cluster-corrected) were found between the two tasks at both sustained and transient levels of processing. One region showed a significant difference in sustained activity between the tasks: the left middle occipital cortex (MOC) was activated more by SJ than TOJ. Investigation of percentage signal change relative to baseline also indicates that on average sustained task-related deactivation was observed during TOJ vs. activation during SJ (sustained main-effect of Task, Table 1 and Figure 4A). During transient events, TOJs revealed significantly more activation than SJs in the middle occipital, middle frontal, precuneus and medial superior frontal regions of the left hemisphere (transient main-effect of Task, Table 1 and Figure 4B). Within the right anterior cingulate there was a significant Task by COA Condition interaction driven by larger deactivations to audio- and video-leading conditions than to PSS and 0 COA, but only during TOJs.

**Table 1:** Significant clusters with peak MNI coordinate, extent, and statistical values.

**Region of peak voxel**	**Hemisphere**	**X**	**Y**	**Z**	**No. Voxels**	**T/F Value**	**Z Score**
**SUSTAINED MAIN-EFFECT OF TASK**							
Middle occipital	Left	−42	−78	31	25	7.25*	4.9
**TRANSIENT MAIN-EFFECT OF TASK**							
Middle occipital	Left	−33	−79	22	149	9.66*	5.66
Middle frontal	Left	−36	8	58	28	9.58*	5.64
Middle frontal	Left	−48	29	34	178	8.78*	5.41
Precuneus	Left	−3	−64	49	31	7.38*	4.95
Sup. medial frontal	Left	−9	26	43	7	6.57	4.64
**TRANSIENT TASK X STIMULUS INTERACTION**							
Anterior cingulate	Left	6	32	−2	9	13.22*	4.69
**TRANSIENT MAIN-EFFECT OF CONDITION**							
Putamen	Left	−24	−4	16	33	15.88*	5.11
Insula	Right	33	23	7	32	13.87*	4.8
Putamen	Right	24	−4	13	16	13.4*	4.72
Angular gyrus	Left	−48	−70	28	12	13.31*	4.7
Superior temporal	Left	−48	−22	7	6	13.3*	4.67
Sup. medial frontal	Right	9	53	16	7	13.02*	4.65
Insula	Left	−30	23	7	10	12.85*	4.62
Superior temporal	Right	63	−34	19	12	12.54	4.57
Anterior cingulate	Left	−3	34	−11	18	12.34	4.53

Clusters showing a voxel-level FWER p < 0.05 based on peak height (Chumbley and Friston, 2009) are indicated by a star (*).

**Figure 5:**
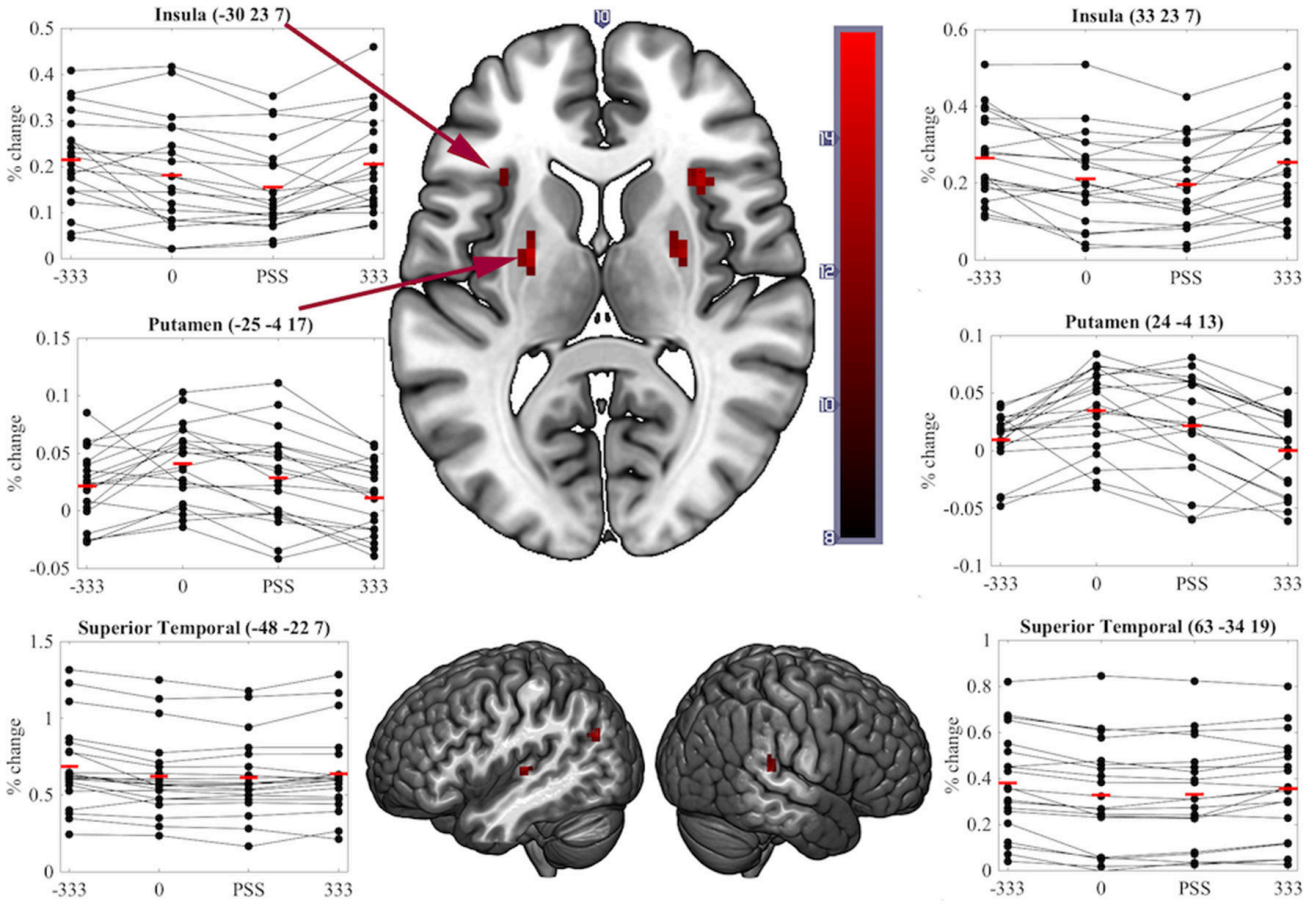
Significant clusters and individual participants' % signal change from the clusters peak voxel for the transient main-effect of COA Condition. % signal change was calculated using a scaling factor of 0.132 (Pernet, 2014). Clusters are presented on axial or sagittal slices of the MNI152_2009bet (Fonov et al., 2011) template using MRIcroGL (www.mccauslandcenter.sc.edu/mricrogl, 12/12/2012).

A significant transient main-effect of COA Condition was found in the bilateral putamen, insula, superior temporal cortex, left angular gyrus and anterior cingulate and right superior medial frontal cortex (Table 1, Figure 5). While our main focus was on effects of task on brain activity, the significant transient main-effect of COA Condition highlights a network of regions involved in processing temporal information in audiovisual stimuli, regardless of the task performed.

No other significant main effects or interactions were found. Since the Group factor (TOJ-able/TOJ-unable) did not produce any significant results, we repeated the above analyses with this factor removed. The results were highly consistent with those described, with the addition that the transient TOJ>SJ effects found in the *left* middle occipital and middle frontal cortex were now also observed in the right hemisphere.

## Discussion

In the current experiment, the same participants made SJs and TOJs to an *identical* set of synchronous and asynchronous audiovisual point-light-drumming stimuli, while their sustained and transient task-related BOLD responses were recorded using fMRI. The results show that, even under identical stimulus conditions, TOJs and SJs have overlapping (main-effect of COA Condition) but divergent neural correlates for both sustained and transient BOLD responses. This neuroimaging evidence is in support of previous behavioral research indicating that the two tasks measure different processes, or aspects, of temporal processing (e.g., van Eijk et al., 2008; Vatakis et al., 2008; Fujisaki and Nishida, 2009; Petrini et al., 2010; Maier et al., 2011; Vroomen and Stekelenburg, 2011; Love et al., 2013). The current transient level results are also largely consistent with the results of a recent study which tested a similar hypothesis using an event-related fMRI analysis and single-event, simpler, audiovisual stimuli (Binder, 2015). In both the event-related results of Binder (2015) and the current transient effects, no region was more activated during SJs than TOJs, and all regions that activated more to TOJs than SJs were in the left hemisphere. Some of these left hemisphere regions, required during TOJs but not, or at least less so, during SJs, were consistent across studies. For example, both found similar MFG clusters, and there is a likely overlap between the cluster labeled as superior/inferior parietal lobule by Binder (2015) and our MOC cluster. While our results and those of Binder (2015) are consistent the current work also suggests that the difference in neural activity between these two tasks is consistent regardless of the stimulus being processed. This is an important and novel finding as it demonstrates that although behavioral performance on both tasks depends on stimulus type/complexity the overall network differences between them do not.

Although we predicted differences in the neural correlates underpinning SJs and TOJs, our design did not preclude finding brain activity common to both tasks. Correspondingly, regions showing sensitivity to COA under both task conditions represent a network for true audiovisual synchrony processing that is independent of task. Notably, we found that COA modulated activity in the bilateral putamen, insula and superior temporal cortex while participants performed either task–all areas previously found to be involved in the processing of audiovisual synchrony either during passive viewing (Calvert et al., 2001), other related tasks (Bushara et al., 2001; Olson et al., 2002) or SJs (Miller and D'Esposito, 2005; Stevenson et al., 2010). As similar regions play key roles in unimodal visual (Davis et al., 2009), auditory (von Steinbüchel et al., 1999), and tactile TOJs (Takahashi et al., 2013), it is possible that these regions represent a network which processes the relative timing of events rather than audiovisual synchrony *per se*. That is, they appear to be modulated by the *relative* timing of events, independent of the stimulated sensory modality or modalities and of the task being performed.

In contrast to transient level results, SJs produced more sustained activity in the left MOC than TOJs. The percentage signal change data actually indicate however that on average this region deactivated during TOJs (Figure 4A). The MOC has previously been found to exhibit task-induced deactivations, i.e., lower BOLD responses during a task than during baseline, and, in addition, these deactivations increase as a function of task difficulty (McKiernan et al., 2003; Hairston et al., 2008). In our previous and current work, we have shown that TOJs are perceived as being more demanding than SJs–a subjective measure that echoes with more objective criteria such as wider TIWs, and larger exclusion rates (Love et al., 2013). Therefore, we propose that the sustained deactivation found in the left MOC is indicative of a reallocation of resources (McKiernan et al., 2003) necessary for an extra stage of processing required during TOJs but not SJs (Jaśkowski, 1993; Binder, 2015; Miyazaki et al., 2016).

During TOJs but not SJs, several regions of the left hemisphere (middle occipital, middle frontal, precuneus and superior medial frontal cortex) displayed increased transient activity compared to baseline. These left-hemisphere results are in line with a voxel-based lesion-symptom mapping (VLSM) study, which argued that regions uniquely involved in *visual* TOJs, as compared to relative size judgments, were lateralized in the left-hemisphere (Wencil et al., 2010). In that study, lesions in both the left inferior frontal and left posterior parietal cortex correlated with visual TOJ deficits. Likewise, lesions of the left hemisphere have also been associated with deficits in *auditory* TOJs (von Steinbüchel et al., 1999; Wittmann et al., 2004). The current experiment and that of Binder (2015) add to these findings by showing that left hemisphere regions were also uniquely involved in *audiovisual* TOJs, even when contrasted with another synchrony-based task. However, it is worth noting that, similarly to Wencil et al. (2010), we are not suggesting TOJs are solely supported by the left hemisphere and SJs by the right hemisphere; indeed, several bilateral regions were activated during both tasks (see Table 1 and Figure 5). Plus, a left lateralized TOJ>SJ effect should be treated with caution since similar effects were also observed in the *right* hemisphere when the Group factor was removed from the current analysis. Furthermore, despite noting that during tactile stimulation the regions activating more to TOJs than SJs were primarily distributed in the left hemisphere, Miyazaki et al. (2016) also found two such regions in the right hemisphere. Comparing unimodal visual TOJs to a shape discrimination task in two experiments, Davis et al. (2009) found bilateral temporal parietal junction activation to be larger for TOJs in the first experiment, while the same effect was found only in the left hemisphere during the second, better controlled, experiment. Taking all this evidence into consideration it appears prudent to talk about a prevalence of left hemisphere regions being more activated for TOJs than SJs rather than about a lateralization of this effect.

Regardless of whether the extra neural responses required to make TOJs are lateralized or not, they do highlight cognitive processing that is over and above that needed to make SJs. Binder (2015) argued that the additional activation was evidence in favor of a two-stage cognitive processing architecture for TOJs (Jaśkowski, 1991), requiring the perception of both (a)synchrony and order–SJs require only the first. Our results are coherent with this argument and further support it by highlighting that *sustained* task-induced deactivation of left MOC may facilitate this extra cognitive processing. This evidence is in opposition to the theory that TOJs can be made using the same information (arrival-time difference between the cues) and cognitive architecture as SJs (Sternberg and Knoll, 1973; Allan, 1975). Comparison of these two tasks using more time-sensitive neuroimaging techniques, such as electroencephalography and magnetoencephalography, would elucidate whether these stages are conducted serially or in parallel. Use of the VLSM technique to search for a double-dissociation between audiovisual TOJs and SJs, similar to that found between visual TOJs and relative size judgments by Wencil et al. (2010), could also greatly increase our knowledge of the overlapping nature of these two processes.

## Limitations

One of the strengths and novelties in our study could also be seen counter intuitively as one of the limitations. As mentioned we used a more ecological and complex stimulus formed by a series of events (nine impact movements and nine resulting sounds) rather than a single, well-defined event (e.g., beep-flash). Having multiple events means that creating the different asynchrony levels between the visual and auditory streams has the effect of realigning sensory inputs at longer COAs. This could have implications and perhaps partly explain the performance of TOJ-unable participants.

However, there are several reasons why we do not consider this a limitation. First of all, it is unclear why this realignment would affect more TOJ than SJ. Considering that participants could have used all the events in both tasks to make their judgments we could assume that the effect of a decrease in asynchrony for some events, due to realignment, should have had an influence on both tasks not only TOJ. Second, we would predict that any influence this realignment had on participants' responses should have been seen for both audio and video-leading conditions. However, this was not the case. Hence, though the effect of auditory and visual event realignment in complex stimuli needs to be considered and discussed we do not believe it undermines the task-related effects presented here. Finally, our interpretation is supported by the high level of consistency between our findings and those of Binder (2015) who used a single-event flash and beep stimulus, for which no realignment at larger audiovisual lags could occur.

Another possible limitation of the current work relates to the relative difficulty of the tasks. In general, when comparing tasks it is prudent to equate difficulty across the tasks. However in reality this is non-trivial, in particular when there is an inherent difference in difficulty between them. Participants in this and our previous work (Love et al., 2013) reported that TOJs were in general more difficult than SJs based on their experience with the tasks for a wide variety of cue onset asynchronies and a wide variety of stimulus types. For simple beep-flash stimuli, for example, we previously (Love et al., 2013) found no quantitative indication of TOJs being more difficult than SJs except for verbal reports, in which 71% of participants thought TOJs were more difficult. This example helps to highlight two different concepts of task difficulty: 1) between-task and 2) within-task difficulty. By within-task difficulty we refer to, for example, performing at a 75% correct level on two different tasks. While difficulty could be believed to be equal in this situation we would argue that this is not necessarily so. For example, it is inherently more difficult to perform at 75% when solving differential equations compared to performing at 75% on a multiplication task. Clearly, there are cases when equating this type of within-task difficulty is not possible. We believe that there is an inherent within-task difficulty difference between SJs and TOJs that cannot easily be equated. Perhaps, as suggested above, this could be due to an extra stage of cognitive processing required for TOJs. That said, it would be informative to compare the two tasks at audio- and video-leading just-noticeable-difference COA levels, as this may be the best control of within-task difficulty. We chose not to do this, as it would lead to different stimulus conditions (COA levels) being presented for each task. In general, behavioral experiments highlighting differences between SJ and TOJ have used identical stimulus conditions to compare the tasks. Here we aimed to investigate the underlying neural mechanisms that reflect the findings of such behavioral work.

As detailed in section Behavioral Results Behavioral Results, there was a significant difference in group mean behavioral performance for the +333 COA condition dependent on whether it was conducted inside or outside the MRI environment. Furthermore, while it was not possible to statistically compare PSS performance from outside and inside the MRI environment it appears that, at least for some participants, performance on the PSS condition was also affected by the MRI environment (Figure 3A). One obvious difference between the experimental procedures in these two situations was the ratio of synchronous to asynchronous conditions presented. In the pre-fMRI experiment multiple asynchronous COA levels were presented whereas during the fMRI experiment an equal number of synchronous (0 COA and PSS) and asynchronous (-333 and +333) COA levels were presented. It is possible that these different experimental contexts influenced behavioral performance. Another possibility is that the noise produced by the MRI scanner made the information from the auditory cue less reliable thus widening the TIW for the participants during the scan, which would result in a lower ability to detect asynchrony particularly when vision led sound.

Our subsample sizes of 9 (TOJ-able group) versus 11 (TOJ-unable group) should be considered as a limitation in the ability to detect differences between the two groups. Indeed no significant differences involving the Group factor were observed. However, our failing to observe significant differences between the groups should not, as with all null results, be interpreted as evidence of no difference. A study designed specifically to test for differences between these two groups involving a larger number of observations per group may well highlight significant differences. Unthresholded statistical maps from the current study (http://neurovault.org/collections/UMJLMEEJ/) indicate, for example, a possible main-effect of Group in bilateral Putamen and in the right STS.

In the current fMRI experiment the visual cue was presented on a screen approximately 65 cm from the head of participants, while the auditory cue was presented via headphones. The relative spatial location of the sensory cues of a multisensory stimulus is one of the main factors in regulating multisensory integration mechanisms (Stein and Meredith, 1993). We have previously shown that for an SJ task, using headphones or speakers placed next to the screen led to no significant difference in behavioral performance when using the same stimuli used in the present study (Petrini et al., 2009a). Therefore, we believe that it is unlikely that this spatial discordance significantly influenced the current results; however, an effect of such spatial discrepancy for TOJs cannot be ruled out. This is a limitation we share with the study by Binder (2015) and it is dictated by the common use of headphones during fMRI studies to reduce background noise. It would be interesting in future studies to ascertain the effect of spatial displacement on both tasks by conducting the TOJ and SJ tasks with both headphones or speakers.

## Conclusion

In conclusion, important differences between the neural correlates of synchrony judgments (SJs) and temporal order judgments (TOJs) have been highlighted at both the sustained and the transient BOLD response levels. The similarity between the current results and those of Binder (2015) provide converging evidence that the divergent neural correlates of these two tasks likely exist regardless of stimulus complexity; however, it is important to also confirm this using contextually natural (not only white dots on a black background) stimuli. We speculate that the more demanding, possibly two-stage, cognitive processing required for TOJs induces a task-induced deactivation of the MOC to reallocate resources to regions required to make the judgment: the middle occipital, middle frontal, precuneus and superior medial frontal cortex. One important conclusion arising from this study is that care must be taken during future attempts to use atypical temporal processing as a diagnostic tool, or to inform the creation of remediation strategies for clinical disorders such as autism and schizophrenia. As SJs and TOJs are underpinned, not only by overlapping, but also by divergent neural mechanisms, atypical processing found for one task may or may not exist for the other (Capa et al., 2014). Neuroimaging studies examining differences in temporal processing between the mentioned clinical populations and the typical developing population could focus on the task unrelated activated regions (e.g., putamen, insula and superior temporal cortex) to identify useful diagnostic markers.

## Data availability

Unthresholded statistical maps were uploaded to NeuroVault.org database and are available at http://neurovault.org/collections/UMJLMEEJ/. The raw data supporting the conclusions of this manuscript will be made available by the authors, without undue reservation, to any qualified researcher.

## Author contributions

All authors participated to experimental design, interpretation, manuscript editing and approval. SL and ML analyzed the data. SL collected the data and wrote the first draft of the manuscript.

### Conflict of interest statement

The authors declare that the research was conducted in the absence of any commercial or financial relationships that could be construed as a potential conflict of interest.
