# Supplementary material for: Overlapping but Divergent Neural Correlates Underpinning Audiovisual Synchrony and Temporal Order Judgments
Source: Front Hum Neurosci. 2018 Jul 3;12:274. doi: 10.3389/fnhum.2018.00274 (PMC6037859; doi:10.3389/fnhum.2018.00274)
Supplement: Supplementary file 4 [file Image_1.PDF]

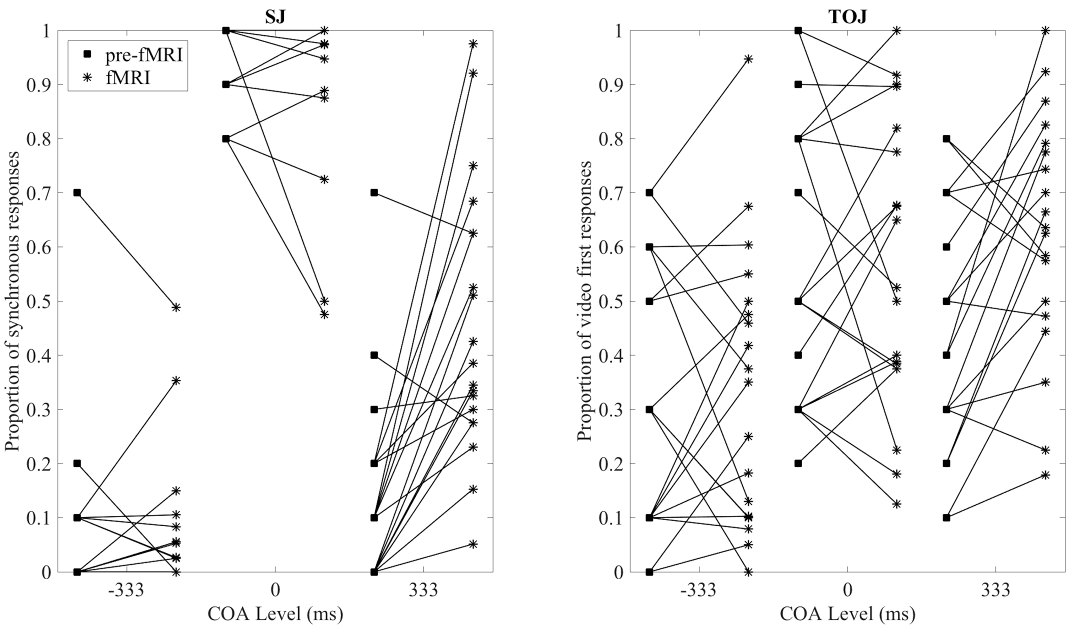

Supplementary Image 1: Pre-fMRI and during fMRI behavioral performance. Proportions of synchronous responses from the SJ task (left) and proportions of video first responses from the TOJ task (right) from individual subjects. Pre-fMRI (filled squares) and during fMRI (stars) proportions from the same participant are connected by black lines.
